# Correlation of inflammatory markers and NFATC4 gene expression among subjects with prediabetes

**DOI:** 10.34172/jcvtr.33272

**Published:** 2024-12-23

**Authors:** Aswathi Rajan, Karpagavel L, Vidya S, Sheena K S, Harilal M D, Deepthi S, Manjusha K, Rachana Raveendran, Ambili P V, Midhun T M, Swathi T, Dinesh Roy D

**Affiliations:** ^1^Chettinad Academy of Research and Education (Deemed to be University), Kelambakkam, Chennai, Tamil Nadu, India; ^2^Department of Biochemistry, Chettinad Academy of Research and Education (Deemed to be University), Kelambakkam, Chennai, Tamil Nadu, India; ^3^Department of Anatomy, Sri Sankara Dental College, Akathumuri P. O, Kerala, India; ^4^Meenakshi Academy of Higher Education and Research (MAHER- Deemed to be University), West K.K Nagar, Chennai, Tamil Nadu, India; ^5^Genetika, Centre for Advanced Genetic Studies, Thiruvananthapuram – 695024, Kerala, India

**Keywords:** Confidence interval, Inflammatory markers, NFATC4 gene expression, Prediabetes, Type 2 diabetes mellitus

## Abstract

**Introduction:**

Prediabetes, characterized by mildly elevated blood sugar levels, significantly increases the risk of developing type 2 diabetes and cardiovascular disease. The condition is linked to higher levels of IL-18, TNF-α, and IL-6, indicating inflammation that may drive type 2 Diabetes Mellitus (T2DM). Despite the known role of inflammation in glucose homeostasis, the involvement of the Nuclear Factor of Activated T Cells 4 (NFATC4) gene in prediabetes remains underexplored. This case-control study aims to investigate the association between physiological, demographic, anthropometric, lifestyle factors, inflammatory markers and NFATC4 gene expression, in the context of prediabetes.

**Methods:**

The study involved 300 participants aged 20 to 50, with 150 diagnosed with prediabetes and 150 healthy controls. After obtaining informed consent fasting venous blood samples were collected for comprehensive assessments, including biochemical, endocrinological and immunological analyses. Specifically, NFATC4 gene expression and inflammatory markers were measured.

**Results:**

The findings revealed significantly elevated levels of IL-18, TNF-α, IL-6, and NFATC4 expression in prediabetic individuals compared to controls. Notably, strong positive correlations were observed between NFATC4 expression and the inflammatory markers. Receiver operating characteristic (ROC) curve analysis identified IL-18 and NFATC4 as the most promising biomarkers for predicting prediabetes, followed by TNF-α and IL-6. Multivariate regression analysis further identified socioeconomic status (SES), IL-18, NFATC4, TSH, triglycerides, and HDL as independent predictors of prediabetes.

**Conclusion:**

These results highlight the key role of inflammation and NFATC4 in prediabetes, stressing the need for strategies to prevent progression to type 2 diabetes and cardiovascular issues.

## Introduction

 Prediabetes, marked by elevated blood sugar levels, greatly increases the risk of developing type 2 diabetes (T2DM) and cardiovascular disease (CVD).^1^ Both the World Health Organization and the American Diabetes Association identify it as a critical public health concern requiring preventive measures.^2,3^ Research links its development to chronic low-grade inflammation, evidenced by increased levels of markers such as IL-18, TNF-α, and IL-6.^4,5^ Additionally, research has identified specific genes associated with diabetes. For instance, changes in the TCF7L2 gene are associated with a higher risk of developing type 2 diabetes.^6^ Prediabetes not only signifies a stage preceding T2DM but also underscores the heightened risk of premature CVD, emphasizing the urgency of preventive measures. Lifestyle interventions, including dietary modifications and increased physical activity, are recommended for individuals with prediabetes to mitigate these risks. Poor diet, lack of physical activity, and obesity are significant risk factors for type 2 diabetes. These factors may contribute to insulin resistance, which is a key characteristic of type 2 diabetes.^7^ However, targeted interventions necessitate a deeper understanding of the molecular pathways involved.

 Despite the recognized role of inflammation in prediabetes, the involvement of specific genes remains underexplored. One such gene of interest is NFATC4, known for its significance in inflammation and glucose homeostasis. This family of transcription factors plays a crucial role in regulating the expression of proinflammatory cytokines and related genes during immune responses.^8^ Dysregulation of Nuclear Factor of Activated T Cells 4 (NFATC4) expression has been linked to prediabetes, although the underlying mechanisms are not fully elucidated. Understanding the relationship between inflammatory markers and NFATC4 gene expression in prediabetes could provide crucial insights into disease mechanisms and potential therapeutic targets.

 India, with its diverse population, faces varying prevalence rates of prediabetes across different regions, with Kerala notably identified as the ‘Diabetes Capital’ due to its high prevalence in the state. Analyzing prediabetes mechanisms in these populations offers vital insights into disease origins and aids in tailoring preventive strategies for the region.^9^ Therefore, this study aims to elucidate the correlation between inflammatory markers IL-18, IL-6, TNF-α and NFATC4 gene expression in prediabetes. By examining these markers in prediabetic individuals compared to controls, we seek to uncover the role of NFATC4 in inflammation associated with prediabetes. The study aims to examine the relationship between physiological, demographic, anthropometric, lifestyle factors, inflammatory markers, and NFATC4 gene expression in prediabetes. The expected outcome is to identify a clear link between elevated inflammatory markers (IL-18, TNF-α, IL-6) and increased NFATC4 gene expression in prediabetic individuals. These findings could guide targeted interventions to improve prediabetes management and prevent T2DM and CVD.

## Materials and Methods

###  Study design

 This case-control study examined the link between inflammatory markers and NFATC4 gene expression in prediabetes. Approved by the Institutional Ethics Committee (08/2022/IECG), all participants gave informed consent. Selecting prediabetic individuals as cases allows for studying the link between inflammatory markers and NFATC4 gene expression in those at risk for diabetes. Healthy individuals were selected as controls to provide a baseline for comparison, which aids in identifying specific changes in inflammatory markers and gene expression associated with prediabetes. This study design facilitates a clearer understanding of the pathophysiological mechanisms that contribute to the progression from prediabetes to diabetes, making it relevant for identifying potential therapeutic targets.

###  Participants

 A total of 300 participants aged 20 to 50 years were recruited for this study. The participants were divided into two groups: individuals with prediabetes and individuals with healthy controls. Each case (prediabetic individual) was matched with one control (1:1 ratio) to enhance statistical power. Prediabetes was defined based on fasting blood sugar levels ( > 125mg/dL: diabetic, 100-125mg/dL: prediabetic). HbA1c levels were measured in all participants ( ≥ 6.5% for diabetes; 5.7% - 6.4% for prediabetes).

###  Setting and locations

 The study was conducted in collaboration with Hridayala Heart and Robotic Research Centre Pvt. Ltd., Thiruvananthapuram, Kerala, and Genetika, Centre for Advanced Genetic Studies. Medical camps were organized during World Heart Day **(September 29)** and World Diabetes Day **(November 14) **for participant recruitment, while laboratory investigations were carried out at Genetika, Centre for Advanced Genetic Studies, Thiruvananthapuram. The study did not include a follow-up period.

###  Eligibility criteria

####  Inclusion criteria

- Participants aged 20 to 50 years.
- Diagnosed with prediabetes based on fasting blood sugar levels (100-125 mg/dL) and HbA1c levels (5.7% - 6.4%).
- Healthy controls with normal fasting blood sugar levels ( < 100 mg/dL) and HbA1c levels ( < 5.7%).

####  Exclusion criteria

- Diagnosed diabetes (fasting blood sugar > 125 mg/dL or HbA1c ≥ 6.5%).
- Chronic inflammatory diseases, autoimmune disorders, or ongoing infections.
- Use of medications affecting glucose metabolism.

###  Sample size 

 The sample size was calculated using the following formula, based on data obtained from various published sources:

 Sample size = Z^2^pq/d^2^

 Where:

 Z = Standard normal deviation

 P = Prevalence

 Q = 1-P

 D = Degree of accuracy.

 By applying this formula, the prevalence **(P)** was derived, providing a reliable estimate based on existing literature and ensuring that the sample size was sufficient for the study.

###  Data collection

 A wide array of information was gathered from all participants, including demographic, clinical, and biochemical data. Socio-demographic information, physical measurements, biochemical parameters, behavioural factors, and family history of diabetes were recorded. Fasting venous blood samples (5-6 ml) were collected from each participant for further analysis after getting ICF. The exposures of interest include demographic factors (age, sex, socioeconomic status), anthropometric measurements (BMI, waist circumference), and lifestyle factors (physical activity, dietary habits) that may influence the inflammatory markers and gene expression.

###  Assessment of demographic and lifestyle parameters

 A comprehensive questionnaire was created, covering key demographic factors such as Sex, Birth Order, Place of Residence, Occupation Type, Age, No: of pregnancy, Duration of married life, Age at marriage, Age of Husband/Wife, Socioeconomic status, and alcohol consumption. The questionnaire was administered through face-to-face interviews with subjects who provided informed consent.

###  Assessment of anthropometric parameters

####  Height

 Height was measured using a stadiometer with a vertical ruler and movable headpiece. The headpiece was adjusted to the top of the individual’s head, and the height was recorded from the ruler.

####  Weight

 Weight was measured using a digital weighing scale placed on a flat surface for accuracy. Subjects stood barefoot and still on the scale, and the stable reading was recorded in kilograms (kg).

####  Body mass index (BMI)

 Body Mass Index (BMI) was calculated using the formula:

 BMI = weight (kg) / height (m)^2^.

####  Abdominal circumference

 Abdominal circumference was measured using a flexible measuring tape. The tape was placed around the abdomen at the level of the navel (umbilicus) while the subject stood upright, ensuring the tape was snug but not compressing the skin. The measurement was recorded in centimeters (cm).

###  Multi-parametric examination of blood samples

 Venous blood samples were analysed for inflammatory markers (IL-6, IL-18, TNF-α) and NFATC4 gene expression. HbA1c levels were included as part of the biochemical assessments to confirm glucose metabolism status.

###  Laboratory investigations

####  Fasting blood sugar

 Fasting blood sugar (FBS) was estimated using the GOD-POD colorimetric method.

####  HbA1C

 HbA1C was estimated using Nephelometry methodology.

####  Cholesterol

 Cholesterol was measured using the Cholesterol Oxidase-Peroxidase (CHOD-POD) methodology.

####  Triglyceride

 The GPO-TOPS method was a commonly used enzymatic method for the quantitative determination of triglyceride levels in biological samples.

####  HDL and LDL 

 HDL and LDL cholesterol was measured using a method calibrated with a cholesterol calibrator.

####  fT4

 The Free T4 assay was a solid-phase, enzyme-labeled chemiluminescent competitive immunoassay.

####  TSH

 The IMMULITE/IMMULITE 1000 Third Generation TSH assay was a chemiluminescent immunometric assay that utilized a solid-phase with two specific binding sites for TSH.

####  Creatinine

 Creatinine concentration was measured using a creatininase enzymatic method, where quinone pigment formation was detected photometrically.

####  Urea and uric acid

 Urea concentration was determined enzymatically by measuring the absorbance of the sample and standard against a standard curve.

####  IL-18, IL-6, and TNF-α

 The assays utilized a sandwich enzyme immunoassay technique with microtiter plates pre-coated with specific antibodies for Human IL-18, IL-6, and TNF-α.

###  RNA extraction

 RNA isolation, cDNA synthesis, Real-Time PCR, and ELISA were performed according to standard protocols. Primers specific to NFATC4 transcripts were designed, and relative NFATC4 expression was calculated using the formula 2^-ΔΔCt^. For total RNA extraction, the RNA Extraction Kit from Origindiagnostics and Research was used. This kit employed a technology based on the guanidine thiocyanate/phenol method combined with a spin column approach. This method eliminated the need for gel-based confirmation. RNA concentration and purity were assessed using a biospectrometer. The primer sequences and their associated data were represented in the following table (Table 1).

**Table 1:** Detailed Primer Specifications for PCR Analysis

**Primer**	**Sequence**	**Length (bp)**	**GC Content (%)**	**Melting Temp (Tm) °C**	**Annealing Temp (°C)**	**MW (g/mol)**	**Purification**
Forward Primer	5'-GCACCGTATCACAGGCAAGATG-3'	22	54.55	62.12	60	6,753.42	HPSF
Reverse Primer	5'-TCAGGATTCCCGCGCAGTCAAT-3'	22	54.55	62.12	60	6,695.35	HPSF

###  Total RNA extraction protocol

 Fresh blood was mixed with Buffer RZ in a 3:1 ratio and incubated at 15-30°C for 5 minutes. After adding 150 μL of chloroform and mixing, the sample was incubated for 3 minutes at room temperature and centrifuged at 12 000 rpm for 10 minutes at 4°C. The separated aqueous phase was transferred to a new tube, mixed with 375 μL of ethanol, placed in a spin column, and centrifuged at 12 000 rpm for 30 seconds. The column was subsequently washed with Buffers RD and RW, dried, and finally, RNA was eluted using 30-100 μL of RNase-free water.

###  cDNA synthesis 

 50 micrograms of RNA was mixed with Oligo (dT)₁₈, random hexamer, dNTPs, and RNase-free water. The mixture was heated at 65°C for 5 minutes, cooled on ice, then combined with RT Buffer and RTase. It was incubated at 25°C for 5 minutes, 50°C for 60 minutes, and inactivated at 95°C for 5 minutes.

###  Primer and master mix quantities in real- time PCR

 To prepare a 20μL PCR mixture, combine 10μL of 2X Real-Time PCR Master Mix, 1μL each of Forward and Reverse Primers (both at 10pmol/μL), 2μL of cDNA, and 6μL of distilled water.

###  Real-time PCR 

 A 20 μL PCR mixture was prepared, containing 2X Real-Time PCR Master Mix, primers, cDNA, and water. The PCR cycling conditions were detailed in the table below (Table 2). The process concluded with a final extension, followed by melt curve analysis.

**Table 2:** PCR Thermal Cycling Conditions

**Step**	**Temperature (°C)**	**Duration**	**Number of Cycles**
Denaturation	94	1 minute	32
Annealing	60	1 minute	
Extension	72	1 minute	
Final Extension	72	10 minutes	

###  Data analysis

 The ΔCT and ΔΔCT values were calculated, and the fold change in gene expression was determined using the 2^-ΔΔCT^ method. The results were then graphically represented using bar graphs.

###  Statistical analysis

 Statistical analysis was performed with Jamovi 2.5.3 and Excel. Quantitative data were reported as mean ± SD or median with interquartile range, while qualitative data were presented as frequency and percentage. Chi-square tests and odds ratios with 95% confidence intervals were used to assess relationships between categorical variables. Continuous variables were evaluated using t-tests or Mann-Whitney U tests. Multivariate binary logistic regression identified prediabetes predictors, with ROC curve analysis and AUC assessing biochemical parameters’ accuracy. A p-value < 0.05 was deemed significant. Potential confounders that were controlled for in the analysis included comorbid conditions (e.g., hypertension, dyslipidemia), medication use, and other factors such as family history of CVD, which may have influenced both the exposures and outcomes. The analysis explored whether certain variables, such as age, sex, and lifestyle factors, modified the relationship between inflammatory markers and NFATC4 expression and the risk of CVD.

## Results

 The study involved 300 participants, aged 20 to 50 years. The participants were divided into two groups: individuals with prediabetes and individuals with healthy controls. The Table 3 presents the distribution of various demographic and occupational characteristics among case and control groups.

**Table 3:** Demographic information presentations

	**Case (n=150)**		**Control (n=150)**		**Total (n=300)**		**χ**^2^	**df**	**p**
	**n**	**%**	**n**	**%**	**n**	**%**			
Sex									
Male	79	52.7	65	43.3	144	48	2.618	1	0.106
Female	71	47.3	85	56.7	156	52			
Birth order new									
1	59	39.3	66	44	125	41.7	0.681	2	0.711
2	44	29.3	40	26.7	84	28			
3 or more	47	31.3	44	29.3	91	30.3			
Residence									
Rural	35	23.3	43	28.7	78	26	2.212	2	0.331
Urban	111	74	100	66.7	211	70.3			
Coastal	4	2.7	7	4.7	11	3.7			
Occupational Type									
Sedentary	71	47.3	66	44	137	45.7	0.336	1	0.562
Nonsedentary	79	52.7	84	56	163	54.3			

The chi-square (χ^2^) test was used to compare the proportions between the groups. A p-value less than 0.05 indicates statistical significance.

Table 4 provides a comprehensive overview of the demographic characteristics of the study population, specifically detailing the sex, birth order, residence, and occupational type of the cases and controls. This information is crucial for understanding the baseline characteristics of our study sample, which is integral to interpreting our findings accurately.

**Table 4:** Comparison of Demographic, Physiological and Lifestyle Characteristics between Case and Control Groups

	**Case**	**Control**	**p**
Age	38.1 ± 6.3	36.9 ± 6.5	0.073
No. of Pregnancy	2(2 - 3)	2(1.75 - 2)	0.075
Duration of Married life (Yrs)	13.4 ± 8.3	13.8 ± 7.7	0.726†
Age at Marriage	26.2 ± 4.7	21.5 ± 9.1	< 0.001
Age of Husband/Wife	39.4 ± 7.5	39.4 ± 9.1	0.951
Abdominal Circumference (cm)	94.5 ± 12.3	94 ± 12.8	0.724
Height (cm)	159.2 ± 11.5	158.7 ± 10.9	0.684
Weight (kg)	69.9 ± 14.5	68.3 ± 13.5	0.323
BMI	21.9 ± 3.8	21.4 ± 3.7	0.333
Blood Sugar (mg/dl)	116.3 ± 8.8	88.1 ± 10.2	< 0.001
Cholesterol (mg/dl)	221.5 ± 41.6	167.3 ± 23.8	< 0.001
Triglyceride (mg/dl)	152.5 ± 38.1	111.9 ± 15.5	< 0.001
HDL (mg/dl)	43.7 ± 8.6	50.1 ± 5.6	< 0.001
LDL (mg/dl)	147.3 ± 34.2	94.9 ± 24.7	< 0.001
HbA1C	5.81 ± 0.4	5.27 ± 0.22	< 0.001
fT4 (pmol/L)	16 ± 5.3	17.1 ± 3.8	0.033
TSH(mIU/L)	4.19 ± 4.06	1.95 ± 1.52	< 0.001
Creatinine (mg/dl)	2.06 ± 1.61	0.75 ± 0.31	< 0.001
urea(mg/dl)	25.7 ± 8.6	17.6 ± 3.9	< 0.001
Uric Acid (mg/dl)	7.45 ± 5.14	4.58 ± 1.06	< 0.001
Birth Order	2(1 - 3)	2(1 - 3)	0.777†

Values were reported as mean ± SD or median (IQR). P-values denoted by † were calculated using the Mann-Whitney U test, while other comparisons were made with independent sample t-tests.

 Individuals with prediabetes had a mean age of 38.1 ± 6.3 years, while controls averaged 36.9 ± 6.5 years.

**Figure 1:**
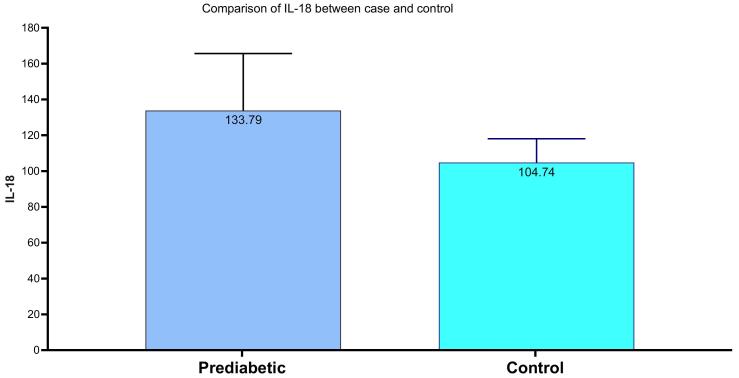
Comparison of IL-18 Levels between cases and controls

 Prediabetic individuals have an average IL-18 level of 133.8 ± 31.9, compared to 104.7 ± 13.3 in the control group. The p-value of < 0.001 indicates a significant difference, suggesting that higher IL-18 levels in prediabetics may reflect potential immune response. (Figure 1)

**Figure 2:**
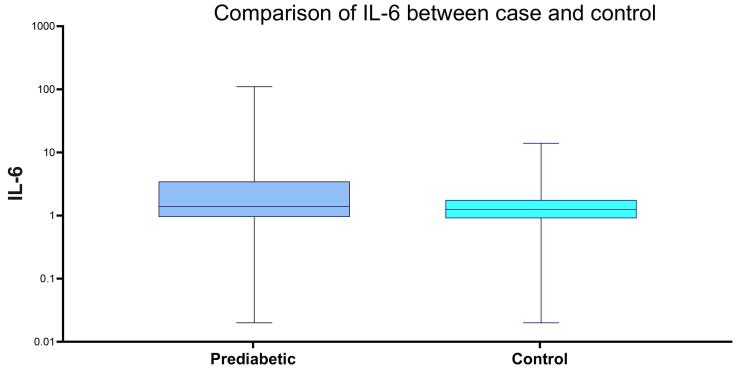
The box plot diagram compares IL-6 levels between prediabetic individuals and controls

Figure 2 represents the middle 50% of data, with the bottom edge (Q1) and top edge (Q3) as the 25th and 75th percentiles, respectively. The median (Q2) is the line inside the box. Whiskers extend to minimum and maximum values for each group.

**Figure 3:**
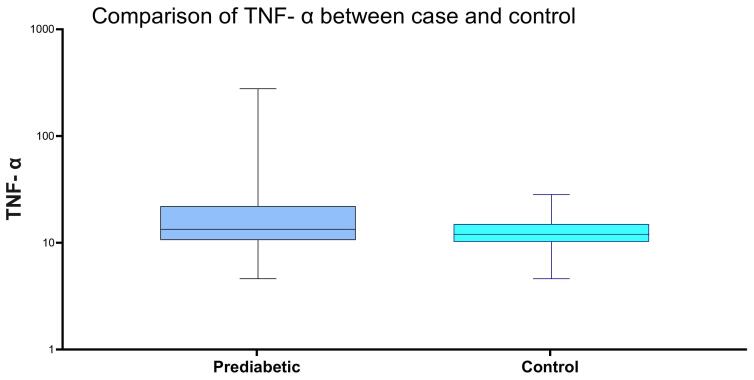
Comparison of Subjects with TNF-α Levels

 The box plot diagram illustrates TNF- α levels in prediabetic individuals and individuals with healthy controls. (Figure 3)

**Figure 4:**
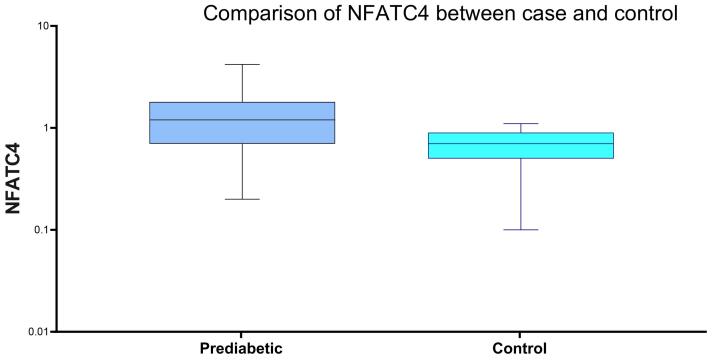
Comparison of Subjects with NFATC4 Levels

 The box plot shows higher NFATC4 levels in prediabetic individuals compared to individuals with healthy controls. (Figure 4)

**Figure 5:**
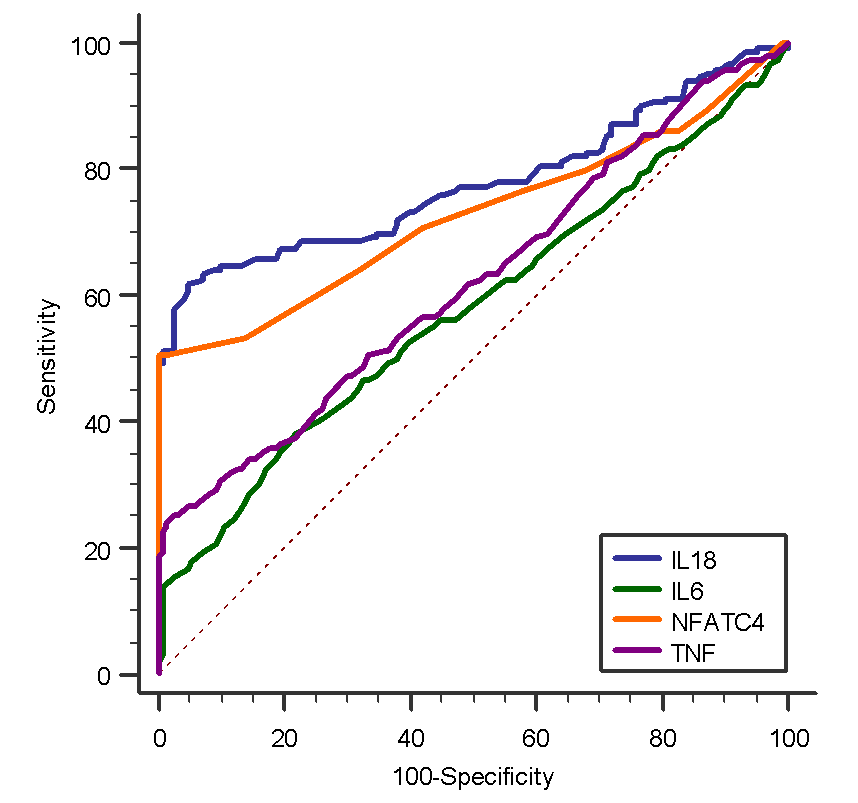
ROC Curve of Inflammatory Markers (IL-6, IL-18, TNF-α) and NFATC4 Gene Expression to predict Prediabetes

 ROC curve analysis evaluated the predictive ability of four inflammatory markers-IL-18, TNF-α, IL-6, and NFATC4-for classifying prediabetes cases and controls. IL-18 exhibited the highest discriminatory power, with an AUC of 0.778, indicating good predictive accuracy. Following IL-18, NFATC4 exhibited a notable ability to discriminate with an AUC of 0.725, suggesting it is an effective predictor. TNF-α had moderate predictive value, with an AUC of 0.622, while IL-6 showed lower discriminatory performance, with an AUC of 0.578. (Figure 5)

 The significance levels (P-values) for all parameters were highly significant, indicating robust discriminatory ability. Sensitivity and specificity varied across parameters, with IL18 showing the highest sensitivity (62%) and TNF-α demonstrating the highest specificity (97.33%). These findings suggest that IL18 and NFATC4 are promising biomarkers for predicting prediabetes, while TNF-α and IL-6 have supplementary roles in risk assessment. (Table 5)

**Table 5:** ROC Curve to classify Cases vs Control

**Variable**	**IL-18**	**TNF- α**	**IL-6**	**NFATC4**
Sample size	300			
Positive group	150			
Negative group	150			
Area under the ROC curve (AUC)	0.778	0.622	0.578	0.725
Standard Error	0.0282	0.0323	0.0331	0.0303
95% Confidence interval	0.727 to 0.824	0.564 to 0.677	0.520 to 0.635	0.671 to 0.775
z statistic	9.876	3.78	2.358	7.437
Significance level P (Area = 0.5)	< 0.001	< 0.001	0.018	< 0.001
Youden index J	0.5733	0.2267	0.16	0.32
Associated criterion	> 124.2	> 21.6	> 1.81	> 0.8
Sensitivity	62	25.33	38	64
Specificity	95.33	97.33	78	68
+ LR	13.29	9.5	1.73	2
-LR	0.4	0.77	0.79	0.53
+ PV	93	90.5	63.3	66.7
-PV	71.5	56.6	55.7	65.4

 The Table 6 presents the results of univariate analysis for the predictors of prediabetes, focusing on various factors, including socioeconomic status (SES), alcohol consumption, and levels of inflammatory markers such as IL-18, NFATC4, TNF-α, and IL-6. For SES, individuals in the upper socioeconomic group were significantly more likely to have prediabetes compared to those in the control group (*P* < 0.001), with an odds ratio (OR) of 2.8 and a 95% confidence interval (CI) ranging from 1.6 to 4.8. Similarly, alcohol consumers had significantly higher odds of prediabetes compared to individuals with healthy controls (*P* = 0.005), with an OR of 2.6 and a 95% CI of 1.3 to 5.3. Elevated levels of IL-18, NFATC4, TNF-α, and IL-6 were all associated with significantly higher odds of prediabetes compared to healthy controls (all *P* < 0.001). The ORs ranged from 2.2 to 33.3, with corresponding 95% CIs indicating a substantial increase in the odds of prediabetes associated with higher levels of these inflammatory markers. Overall, these findings suggest that upper socioeconomic status, alcohol consumption, and elevated levels of inflammatory markers are significant predictors of prediabetes, highlighting their potential role as risk factors for the development of this condition.

**Table 6:** Univariate analysis of subjects based on demographic, lifestyle and inflammatory markers

	**Case**		**Control**		**Total**		**p**	**OR**	**95%CI for OR**
	**n**	**%**	**n**	**%**	**n**	**%**			
SES									
Upper	52	34.7	24	16	76	25.3	< 0.001	2.8	1.6 - 4.8
Lower	98	65.3	126	84	224	74.7			
Alcohol consumption									
Yes	30	20	13	8.7	43	14.3	0.005	2.6	1.3 - 5.3
No	120	80	137	91.3	257	85.7			
IL-18									
> 124.2	93	62	7	4.7	100	33.3	< 0.001	33.3	14.6 - 76.2
≤ 124.2	57	38	143	95.3	200	66.7			
IL-6									
> 1.81	57	38	33	22	90	30	0.002	2.2	1.3 - 3.6
≤ 1.81	93	62	117	78	210	70			
TNF- α									
> 21.6	38	25.3	4	2.7	42	14	< 0.001	12.4	4.3 - 35.7
≤ 21.6	112	74.7	146	97.3	258	86			
NFATC4									
> 0.8	101	67.3	55	36.7	156	52	< 0.001	3.6	2.2 - 5.7
≤ 0.8	49	32.7	95	63.3	144	48			

OR, Odds Ratio; CI, Confidence Interval

 The multivariate analysis reveals significant associations between certain factors and outcomes. Higher socioeconomic status (SES) is notably linked with increased odds of the outcome (*P* = 0.006, Adj. OR = 5.1, 95% CI: 1.6 - 16.4). Elevated interleukin-18 (IL-18) levels are strongly associated with higher odds of the outcome (*P* < 0.001, Adj. OR = 44.3, 95% CI: 11.0 - 178.8). Additionally, levels of thyroid-stimulating hormone (TSH), triglycerides, and high-density lipoprotein (HDL) show significant associations with the outcome (*P* < 0.001 for all). Conversely, alcohol consumption and levels of interleukin-6 (IL-6), and tumour necrosis factor-alpha (TNF-α) do not demonstrate significant associations. These findings underscore the importance of SES, IL-18, NFATC4, and lipid profile markers as potential predictors of the outcome. (Table 7)

**Table 7:** Multivariate regression model of predictors of Prediabetes

	**B**	**S.E.**	**Wald**	**df**	**p**	**Adj.OR**	**95%CI for Adj. OR **
SES (ref- Lower)	1.6	0.6	7.7	1.0	0.006	5.1	1.6 - 16.4
Alcohol consumption (ref- non-alcoholic)	0.0	0.8	0.0	1.0	0.993	1.0	0.2 - 4.9
IL-18 (ref- ≤ 124.2)	3.8	0.7	28.3	1.0	< 0.001	44.3	11 - 178.8
IL-6 (ref- ≤ 1.81)	0.7	0.6	1.1	1.0	0.284	1.9	0.6 - 6.3
TNF- α (ref- ≤ 21.6)	1.1	0.9	1.4	1.0	0.241	2.9	0.5 - 16.8
NFATC4 (ref- ≤ 0.8)	2.1	0.6	11.7	1.0	0.001	7.8	2.4 - 25.6
TSH(mIU/L)	0.4	0.1	12.1	1.0	0.001	1.5	1.2 - 1.9
Triglyceride (mg/dl)	0.1	0.0	35.3	1.0	< 0.001	1.1	1.1 - 1.1
HDL (mg/dl)	-0.3	0.0	28.9	1.0	< 0.001	0.8	0.7 - 0.8

OR, Odds Ratio; CI, Confidence Interval

**Figure 6:**
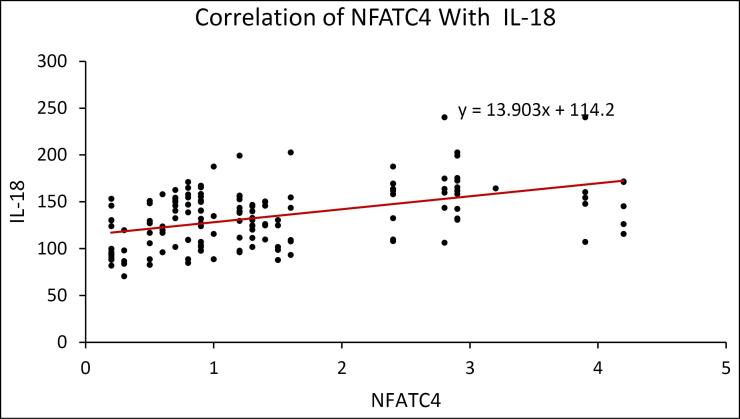
Scatter Plot Showing Correlation of NFATC4 with IL-18

**Figure 7:**
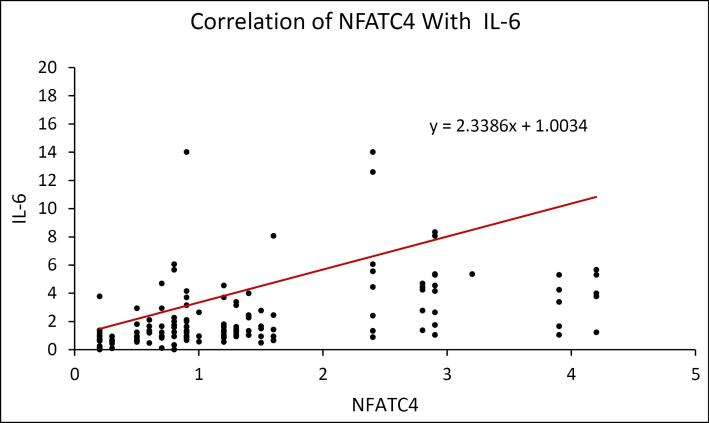
Scatter Plot Showing Correlation of NFATC4 with IL-6

**Figure 8:**
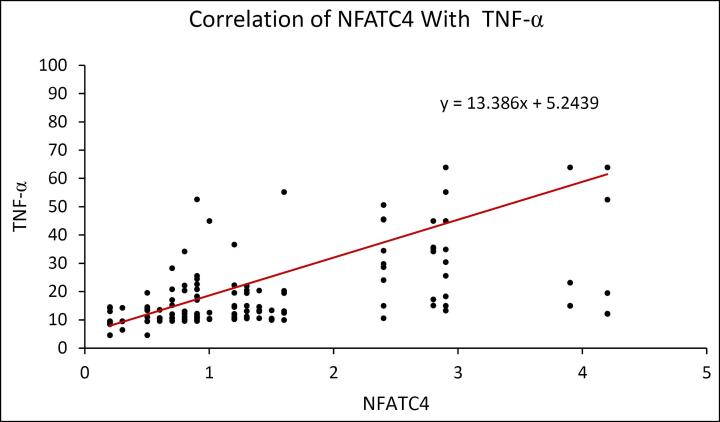
Scatter Plot Showing Correlation of NFATC4 with TNF-α

 The scatter plot reveals that IL-18 has a positive correlation with NFATC4. (Figure 6) The scatter plot shows a positive correlation between IL-6 and NFATC4. (Figure 7) The scatter plot reveals that TNF-α has a positive correlation with NFATC4. (Figure 8)

## Discussion

 Prediabetes represents a critical stage in the progression towards type 2 diabetes mellitus (T2DM), making the identification of risk factors essential for early intervention and prevention strategies. This case-control study explored a range of demographic, physiological, lifestyle, biochemical, inflammatory, and genetic parameters to identify predictors of prediabetes development. It was found that higher levels of inflammatory markers (IL-18, TNF-α, IL-6) and increased NFATC4 gene expression were associated with a greater risk of prediabetes compared to healthy controls.

 In addition to these inflammatory and genetic markers, significant predictors included age at marriage, SES, alcohol consumption, blood sugar, cholesterol, triglyceride, HDL, LDL, HbA1C, fT4, TSH, creatinine, urea, and uric acid were considered to be statistically significant factors. Variables like age, number of pregnancies, duration of married life, age of husband/wife, abdominal circumference, height, weight, BMI, fT4, and birth order were found to be non-significant as shown in Table 4. The combination of elevated inflammatory markers, NFATC4 expression, and lifestyle factors like SES and alcohol consumption provides a comprehensive view of prediabetes risk.

 IL-18, IL-6, and TNF-α were selected as the focus of this study due to their strong association with the chronic inflammation underlying prediabetes and its progression to cardiovascular disease. These cytokines are key indicators of metabolic dysfunction, making them more relevant to the study objectives. This selection allows for a more targeted investigation of the role of NFATC4 in these critical processes.

 The study found a significant difference in mean age at marriage between the two groups: 26.2 ± 4.7 years and 21.5 ± 9.1 years (*P* < 0.001), as shown in Table 4. Zhang et al revealed that prediabetes raises the risk of cardiovascular disease and mortality, especially in younger individuals, with its impact declining as age increases. Additionally, younger age at marriage has been associated with a higher risk of developing prediabetes due to lifestyle changes such as dietary habits and physical activity.^10^

 Robbins et al demonstrated that higher income, education, and occupational status were associated with a lower risk of diabetes in women, though these associations weakened after adjusting for lifestyle factors.^11^ Qi et al similarly found that low income was strongly associated with prediabetes, but this link lost significance when adjusting for other socioeconomic indicators.^12^

 In contrast, the present study revealed that individuals in the upper socioeconomic bracket had a notably higher likelihood of prediabetes compared to controls (OR = 2.8, 95% CI: 1.6 to 4.8, *P* < 0.001), as shown in Table 6. Multivariate analysis revealed that higher socioeconomic status (SES) was associated with a greater likelihood of prediabetes (*P* = 0.006, adjusted OR = 5.1, 95% CI: 1.6 to 16.4), underscoring the complex relationship between SES and prediabetes risk, as detailed in Table 7.

 The association between alcohol consumption and prediabetes risk, noted by Cullann et al and Park et al highlights the need to address alcohol intake as a modifiable risk factor for prevention.^13,14^ Despite the lack of significant associations in multivariate analysis, the current study’s finding of heightened prediabetes risk among alcohol consumers aligns with these observations.

 Smith et al highlighted that 23.9% of the cohort exhibited prediabetes, characterised by elevated fasting blood glucose (FBG) despite normal haemoglobin A1c (HbA1c) levels, suggesting a potential correlation between elevated FBG and increased health risks.^15^ Jeon JY et al established that HbA1c is an acceptable complementary diagnostic tool for diabetes.^16^ As mentioned in Table 4, blood sugar levels were significantly higher in the cases (116.3 ± 8.8 mg/dl) compared to the controls (88.1 ± 10.2 mg/dl) (*P* < 0.001). Furthermore, HbA1c levels were notably elevated in the cases (*P* < 0.001), highlighting the importance of monitoring both fasting blood glucose (FBG) and HbA1c for the early detection and management of prediabetes and diabetes.

 In the present study, Table 4 indicated that cholesterol, triglyceride, and LDL levels were significantly elevated in the case group compared to controls (*P* < 0.001 for all), consistent with Dinarvand et al and Jasim et al who emphasised the predictive value of these markers for prediabetes and T2DM. This evidence underscores the clinical significance of lipid and glycemic markers in assessing prediabetes risk.^17,18^

 Qais et al at General Mosul Hospital found that “elevated serum creatinine levels were significant markers of kidney function impairment in Type 2 DM patients, especially in prediabetes cases,” while Shirsath et al indicated that “elevated uric acid levels in male type 2 diabetes patients indicate a higher prediabetes risk.”^19,20^ Kodama et al’s meta-analysis established a direct link between “serum uric acid levels and the risk of type 2 diabetes.”^21^ In the present study, as highlighted in Table 4, significantly higher levels of creatinine, urea, and uric acid were observed in the case group compared to controls (all *P* < 0.001), underscoring the need for early detection and intervention to prevent diabetes-related complications.

 Chaker et al linked high TSH levels to increased type 2 diabetes risk and high fT4 levels to reduced risk.^22^ Liu et al associated low TSH levels and a higher fT3/fT4 ratio with greater prediabetes risk.^23^ As shown in Table 4, the present study supports these findings, with the case group displaying lower fT4 levels and higher TSH levels compared to the control group, suggesting a potential association between thyroid function and prediabetes risk.

 Gateva et al indicate that chronic low-grade inflammation, marked by elevated levels of cytokines like IL-18 and IL-6, contributes to prediabetes. Studies indicate that IL-18 and IL-6 levels are higher in prediabetic individuals than in healthy controls.^24^ Huang et al found that IL-6 levels are significantly higher in prediabetes, indicating a potential link to type 2 diabetes onset.^25^ Mirza et al and other studies report increased IL-6 and TNF-α levels in individuals with diabetes.^26,27,28^ In this study, inflammatory cytokines IL-18, IL-6, and TNF-α showed significant differences between cases and controls (*P* < 0.001), suggesting their association with prediabetes. IL-18 had the highest discriminatory power with an AUC of 0.778, followed by NFATC4 with an AUC of 0.725. TNF-α and IL-6 also showed predictive capabilities with AUCs of 0.622 and 0.578, respectively, as detailed in Table 5. Elevated IL-18, IL-6, and TNF-α levels were associated with significantly increased prediabetes odds in univariate analysis (all *P* < 0.001). Notably, IL-18 exhibited the highest odds ratio (2.2 to 33.3), as detailed in Table 6. As outlined in Table 7, multivariate analysis confirmed IL-18 as a strong independent predictor (*P* < 0.001, Adj. OR = 44.3), while IL-6 and TNF-α were insignificant. NFATC4 levels were significantly lower in controls, with an AUC of 0.725. Increased NFATC4 levels were associated with higher prediabetes risk (OR 2.2 to 33.3, *P* < 0.001) and an 11.7-fold increase in multivariate analysis (*P* = 0.001), as in Table 7. Scatter plots indicated a positive correlation with inflammatory markers, as illustrated in Figures 6 to 8. These findings highlighted IL-18 and NFATC4 as significant predictors of prediabetes. However, limitations include a non-representative sample and geographical constraints, affecting the generalizability of the study results.

## Conclusion

 In conclusion, elevated IL-18, NFATC4, TNF-α, and IL-6 levels suggest inflammation is key in prediabetes pathogenesis, highlighting the need for further research into interventions to delay or prevent type 2 diabetes. Implementing lifestyle changes in prediabetic individuals can prevent diabetes, improve glycemic control, reduce cardiovascular risks, and save healthcare costs while enhancing public health. Nevertheless, the study highlights the necessity to advance detailed study into the same.

## Acknowledgements

 We sincerely appreciate the support and resources provided by Chettinad Academy of Research and Education (Deemed to be University), Kelambakkam, Chennai, Tamil Nadu, India, and Genetika, Centre for Advanced Genetic Studies, Thiruvananthapuram, Kerala, India.

## Competing Interests

 All authors declare that they have no conflicts of interest.

## Ethical Approval

 Ethical approval (08/2022/IECG) was secured from the Institutional Ethics Committee of Genetika.
